# The link between English foreign language teacher’s professional identity and their critical thinking that leads to teacher’s success in the Chinese context: Leaders motivational language as a moderator

**DOI:** 10.3389/fpsyg.2022.983215

**Published:** 2022-08-12

**Authors:** Fangfang Ding, Xingyu Liu, Alaa Amin Abdalla, Muhammad Latif Khan, Fouzia Akram

**Affiliations:** ^1^College of Humans, Sichuan Agricultural University, Ya’an, China; ^2^Academic Programs for Military Colleges, Abu Dhabi University, Al Ain, United Arab Emirates; ^3^Global College of Engineering and Technology, Muscat, Oman; ^4^Department of Business Administration, University of Prince Mugrin, Madina, Saudi Arabia

**Keywords:** professional identity, critical thinking, teacher success, leader motivational language, English Teacher, MNC’s School

## Abstract

The purpose of this study is to examine the link between English foreign language teacher’s professional identity and employee success via mediating role of critical thinking. Further, we examined the moderating role of leader motivational language between employee professional identity and critical thing and also indirect effect on employee success via critical thinking. We collected data from Chinese MNC’s school by using time lagged study design. We used hierarchical linear regression for direct hypotheses and Hayes PROCESS model’s for mediation, moderation, and mediation moderation analysis. The results show that there is positive relation between employee’s professional identity and teacher’s success. Further, critical thinking mediates the link between professional identity and employee success. The results of the moderated mediation analysis show that critical thinking mediated the interaction of employee’s professional identity and leader’s motivational language on teacher’s success.

## Introduction

Studies on Chinese Multinational Corporation (MNC’s) schools employee’s and their success have been examined in recent years as part of research pertaining to employee’s professional development. Studies on professional identity provide insights regarding the factors that influence a person’s proclivity for a certain chosen profession (Grove et al., 2022). Professional identity is not a fixed entity but rather a dynamic one that changes over time-based on how we see ourselves, how others perceive us, and how society views us (Tripathi et al., 2020). Empirical evidence shows that what we name ourselves and how we express this, are significant factors in determining our professional identity (Makovec, 2018; Wang et al., 2020). Even more significant is that we take control of our careers by investing in ourselves via professional development. This may be the defining characteristic of someone who considers themselves professional. According to Brewer and Roccas (2001) individuals strive to construct their identities based on their personal characteristics (individual), bilateral connections (relational), and group affiliations (collective). Employees’ sense of “occupational or professional identity” is an important part of their self-identification (Shim et al., 2009). Developing a professional identity opens the way for a career to be recognized as a definitive and significant contribution to the employee success. Professional identity composition is considered “a process of interplay between multiple contexts and the individual (Sheybani and Miri, 2019).” Among the most important factors in determining one’s sense of identity are relationships with other people from political, social, and cultural contexts (Demo and Hughes, 1990). Several studies have indicated that an employee’s professional identity is associated with various factors, including their ability to change, effectiveness, confidence, dedication, motivation, and happiness in their teaching career (Danielewicz, 2001; Ng and Feldman, 2008). There is a growing concern regarding how Chinese MNC’s schools employees feel about themselves and their professional identity (Noronha and D’Cruz, 2009). This highlights the need for more research on the impact of employee’s identities on the professional growth of their careers (Eva et al., 2020). Chinese MNC’s schools employees worry about their professional lives because they have to keep up with the pace of change and the growth of knowledge throughout their careers (Quintanilla and Ferner, 2003; Duţă and Rafailă, 2014).

In the workplace contexts, professional identity is reflected as important construct that creates a balance between professional self-image and the perceived responsibilities perform at work (Sutherland et al., 2010). Fogle and Moser (2017) also stated that gaining insight into employee’s decision-making processes, attitudes, and beliefs requires an awareness of employee’s various identities. Perhaps employee’s success might be attributed to their professional identity (Lonnemann et al., 2021). As a result, organizations should look at how employees see their professional identities and the factors that contribute to employee success.

In addition, this study aimed to investigate the extent to which Chinese MNC’s schools employee’s professional identities linked to their levels of critical thinking. Critical thinking is defined as “learning how to ask and answer questions of analysis, synthesis, and evaluation,” and this study sought to determine whether or not such a link exists (Hashemi and Ghanizadeh, 2012). In this context, critical thinking involves two interrelated components: recognizing and questioning assumptions, as well as visualizing and analyzing the perspectives of others (Spector and Ma, 2019). It should come as no surprise that employees first need to demonstrate their capacity for critical thinking before effectively instruct employees in this skill (Dekker, 2020). Although the cultivation of this talent is very important for employees to be success. When employees apply critical thinking, they don’t just accept everything that may happen or every argument that could be made (Boer et al., 2018). On the other hand, employees are more prone to cast doubt on every conceivable consequence and line of reasoning. Employees are interested in investigating the matter more in-depth and seeing evidence of the potential solutions and outcomes to be successful.

The ability to think critically is not something that comes naturally (Puccio et al., 2006). They are created through the course of time and experience and by leader’s motivational language (Avolio and Vogelgesang, 2012). Leaders can persuade their organizations to follow a top-down approach by using language to express management goals, vision, incentives, strategic planning, and organizational activities (Mansaray, 2019). Such motivational language play a significant role in employee professional development (Heitz-Mayfield et al., 2018). Employee professional identity may be strongly influenced by the underlying motivating elements in speech messages that leaders’ language and communication abilities convey (Ling and Guo, 2020). Leaders’ efforts to assist their workers comprehend the significance and duties of their work, to promote initiatives, critical thinking and to help employees receive emotional support and success may be some of the specific motivating components of their leadership for employees success (Dirani et al., 2020). Subordinates would probably agree with the motivational language of leaders who can build their professional identity and mutual understanding with their subordinates in the workplace (Tao et al., 2022). As employees get more emotionally invested and understand one other, their success and conduct improve.

**FIGURE 1:**
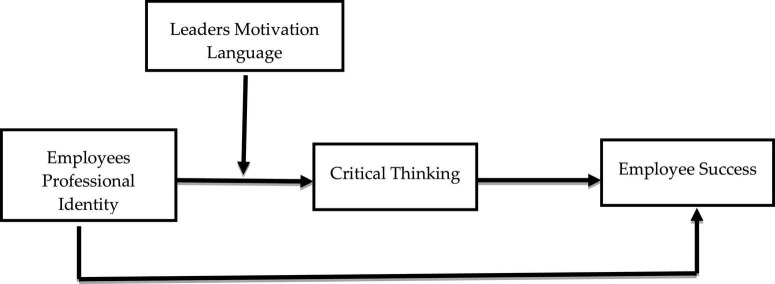
Hypothesized moderated mediation model.

Hackworth et al. (2018) argues that employees must first be taught how to build critical thinking abilities and leadership motivating language gives them the guts to utilize them in their professional careers to achieve success. Motivational Language used by leaders and the attitudes they project are key success factors for employee’s engaged in developing instructional materials and raising educational standards. It was stated by Khany and Fakhar Shahreza (2016) that if employees improved their critical thinking abilities, it would have a major impact on their performance. Therefore, this research aims to examine this evidence in the context of Chinese MNC’s schools by investigating the connection between employee’s professional identities and the level of success they achieve via the use of critical thinking (Figure 1). This may provide Chinese MNC’s schools employees with ideas for developing their critical thinking abilities and the quality of professional identity with the end goal of improving the performance in the Chinese MNC’s schools. In addition, data based on professional identity, critical thinking, leaders’ motivating language, and employee’s success helps MNC, s to assess their current and future challenges.

## Literature review and hypothesis development

### Professional identity and teacher success

The professional identity is the framework through which an employee articulates their professional qualities (Lasky, 2005). According to Sardabi et al. (2018), the term “professional identity” refers to “the attitudes, values, knowledge, beliefs, and abilities shared with others within a professional community.” An employee’s professional identity is connected to an individual’s identity and self-concept that emerges from an employee’s comprehension of and reflection on his or her career-related experiences (Wang et al., 2020). This understanding and reflection is what gives rise to an employee’s professional identity (Vabo et al., 2021). Employees’ professional identities are shaped in various ways, including historically, culturally, and socially, and are influenced by various circumstances (Wei, 2021). Professional identity in the workplace is based on three principles: (1) Specialization-related abilities; (2) one’s moral integrity; and (3) one’s KSA’s ability (Horner et al., 2005).

Professional identity plays a key role in teacher success. The employee’s professional identity level is shown and may be used to measure an employee’s level of success (Khan et al., 2022a). Many academics in the field of organizational research have focused on the question of what characteristics contribute to the success of employees (Zada et al., 2022b). In the previous studies that focused on identifying characteristics of employee success (Celsi and Gilly, 2010), Brosh (1996) identified the primary characteristics of successful employees as being skilled, maintaining sufficient information regarding their job roles, being a good teammate, and being capable of providing good results. Furthermore, according to Tamblyn et al. (2000), successful workers are innovative, have adequate subject knowledge, provide positive encouragement to the organization, be adaptable and a risk-taker, respect their fellow employees, have a sense of humor and lastly be happy of their employment. Subsequently, there are many qualities that Goodson (2003) suggests highly productive workers should have, including a calling to their career, professional knowledge required, personal attributes such as empathy and the ability to be an effective employee.

**Hypothesis 1**: There is positive association between employee’s professional identity and teacher success.

### Mediating role of critical thinking

According to Khany and Fakhar Shahreza (2016), employees’ professional identities are linked to their critical thinking abilities. As a consequence of the research, it was found that the ability of workers to think critically, build a professional identity, and then succeed in their careers were all closely linked. In addition, their findings revealed that the factors studied were intricately intertwined. It was also shown that higher-order thinking abilities (analytical thinking, critical thinking) and self-monitoring positively impacted employee performance, as reported by Bagheri and Ghanizadeh (2016). All of the components of critical reflection were shown to have a favorable effect on employee success (Lyubomirsky et al., 2005). In the job, critical thinking is essential. Critical thinking, after all, helps employees solve issues and devise tactics that help them succeed in their careers (Halpern, 2013). As a result, organization may want to recruit individuals with a high level of critical thinking ability (Varenina et al., 2021). When faced with a particular issue, those who use critical thinking use logic to figure out what to do or believe (Renatovna and Renatovna, 2021). Critical thinkers are self-aware, self-reflective, and capable. With critical thinking, you can link concepts rationally, analyses and assess arguments, uncover flaws in your or others’ work, solve complicated issues, and reflect on your actions and thoughts.

A person capable of critical thinking is not just good at gathering knowledge but also understands how to utilize that information to conclude significant facts and consequences (Warsah et al., 2021). People capable of critical thinking are superior problem-solvers than those who merely remember knowledge because critical thinkers can conceptualize potential outcomes (Frykholm, 2021). As a result, organizations place a high emphasis on critical thinking, particularly in positions where the development of strategy is vital (Parra et al., 2021). The ability to think critically is categorized as a “soft talent,” which indicates that it is a trait that comes naturally to an individual. It is not impossible to improve in this area (Browne, 2021). People can better understand themselves and their motives, as well as their path to success, using critical thinking (Sari and Prasetyo, 2021). Suppose you can draw conclusions from information to determine which aspects are the most significant and then apply those to your life (Changwong et al., 2018). In that case, you will be able to improve your circumstances, enhancing your general happiness, fulfillment, and success.

**Hypothesis 2:** Critical thinking mediates the link between professional development and teacher success.

### Leader motivational language as a moderator

Language that is inspiring and encouraging is essential for effective leadership communication. The term “motivating” refers to the ability of the spoken words of a leader to foster high levels of motivation in the workers toward the critical thinking of the employees and the success of the employees (Rabiul and Yean, 2021). The development of leader motivational language has its origins in various motivational theories, such as the model of job characteristics (Piccolo and Colquitt, 2006; Oldham and Fried, 2016), the theory of goal setting (Locke and Latham, 1990), the model of task- and people-oriented leadership (Yukl, 2012), and the sense-making theory (Weick, 1995). “Direction-giving language,” “meaning-making language,” and “empathe language” are all terms coined by Sullivan et al. (1988) to describe three critical types of leadership speech acts that promote critical thinking among employees. These are: (1) Communicating information to reduce uncertainty, (2) creating organizational meaning through employee mental models, and (3) establishing emotional bonds with employees through social reciprocity and trust.

First, direction-giving language represents a leader’s desire to decrease ambiguity by encouraging employees to establish their professional identities and preparing them to apply their thinking capacity to achieve their goals successfully (Siyal et al., 2021b; Saeed et al., 2022). This is accomplished by assisting employees (Wikaningrum and Yuniawan, 2018; Zada et al., 2022a). Therefore, the main aspects of direction-giving language are related with the idea of goal setting (Locke and Latham, 1990) and the paradigm of task-oriented leadership (Yukl, 2012). Second, leaders’ use of language that creates meaning encourages their subordinates to consider how they may communicate the organization’s objectives, culture, mental models, work values, and the importance of their tasks orally in order to be successful (Wikaningrum and Yuniawan, 2018; Siyal et al., 2020). The process by which leaders influence how their followers view their duties and the relevance of their labor for the focus organization is reflected by the linguistic meaning as a system of interactive symbolism (Oldham and Fried, 2016; Siyal and Peng, 2018). Motivating people to attain high levels of job performance and success requires considering a number of important job design factors, including the meaningfulness and value of the work they do (Oldham and Fried, 2016). Leaders’ oral communications may better instill a feeling of duty and purpose in their followers when they use language that makes sense (Gutierrez-Wirsching et al., 2015). The motivational underpinning of this language is developed from the paradigm of work characteristics (Oldham and Fried, 2016) and sense-making theory (Weick, 1995). In conclusion, using empathetic language in oral communication aims to develop emotional connections between leaders and followers. Leaders utilize empathetic language to support and aid followers in a variety of professional circumstances (Kock et al., 2019). Followers who lack emotional links with their organizations perceive that their organizations care for them and worry less about them; as a result, followers are less inclined to take the initiative to work hard (Nielsen et al., 2008; Siyal et al., 2021a). Leaders use strategies to control their emotions successfully will assist their staff in terms of their wellbeing and proactive conduct (Schraub et al., 2014). Through the use of critical thinking approaches, the symbolic interactionist approach and the role identity theory indicate that pleasant social interactions enhance workers’ personalities and help them build their professional identities at work (Burke and Tully, 1977; Zada et al., 2022b). People’s professional identities are built and maintained via interactions with relevant individuals (Lamb and Davidson, 2005; Khan et al., 2020). Leadership language is an important context component that affects workers’ sense of professional identity; hence, employees’ relationships with their leaders are critical to success (Hu et al., 2018).

**Hypothesis 3:** The positive connection between professional identity and critical thinking is moderated by the leader’s motivational language.

**Hypothesis 3a** The strength of the mediated relation between employees professional identity and teacher success (through critical thinking) will be high when the leader motivational language is high (vs. Lower).

## Materials and methods

### Participants

The data was collected from 274 employees employed in the Chinese MNC’s school through time-lagged design and convenience sampling. The data regarding professional identity, critical thinking and leader motivational language was taken from employees and teacher success was from students. The data were collected in three phases. The participants were selected for inclusion according to their willingness to participate in the research. Participants were informed of the study’s overall goal and reassured that the data they provided would be kept strictly secret and used solely for the purposes of this particular study. At time 1, the data was collected regarding Geographic’s and employees’ professional identity (89.47%). At time 2, the data were collected from leader motivational language (87.23%). At time 3, the data on critical thinking and teacher success were collected (84.71%). The demographic analysis is as follows: male respondents were 77.23 and 35.71 years were average. 87.41% had a master’s degree or above. The respondents were chosen based on their willingness to take part in the study. The general aim of the study was also explained to the participants, and they were reassured that their information would remain confidential and be utilized only in the present study research.

### Instruments

#### Employee professional identity

Professional identity of employees was measured by using Wei (2008) 18-items scale that consists four sub dimensions: occupational values, role value, the sense of occupational belonging, and professional behavior inclination. In this study, the Cronbach’s alpha coefficient of this scale was 0.87.

#### Leader motivating language

Leader motivational language was measured by using Mayfield and Mayfield (2007) 9-items scale. This scale has three sub scale (1) direction-giving language sample item is “My supervisor gives me clear instructions about solving job related problems.” (2) Empathetic language, such as, “My supervisor shows me encouragement for my work effort.” (3) Meaning-making language, such as “My supervisor offers me advice about how to behave at the organization’s social gatherings.” Cronbach’s alphas for direction-giving language, empathetic language, and meaning-making language were 0.84, 0.78, and 0.83, respectively.

#### Critical thinking

Employee’s critical thinking was measured by using 25 items scale developed. Sample item is “After reading it, I check important information, even if it seems to be true.” In this study, the Cronbach’s alpha coefficient of this scale was 0.85.

#### Teacher success

Employee success was rated by supervisors and measured by scale developed by Williams and Anderson (1991) 7-items. In this study, the Cronbach’s alpha coefficient of this scale was 0.84. A sample item is “Engages in activities that will directly affect his/her performance.”

### Data analysis

#### Confirmatory factor analysis

We used AMOS (Muthén and Muthén, 2010) to perform a series of confirmatory factor analyses on our survey measures to confirm our survey measures’ factor structure. We included the variables under examination in the tests to fulfill the suggested parameters’ requirement to sample size ratio for estimation (1:5) (Bentler and Chou, 1987). Among performing different episodes of CFA, the four-factor model was best fit: χ^2^/df = 2.56, χ^2^ = 152.42, *p* < 0.001, SRMR = 0.04, TLI = 0.90, CFI = 0.91, RMSEA = 0.05, GFI = 0.91, as shown in Table 1. The value of RMSEA was less than 0.10; the values of GFI, IFI, CFI, and TLI were higher than 0.90; and the value of χ^2^/df was less than 3, which indicated that the four constructs in this study had acceptable discriminant validity and represented the four different constructs well. Now we can test the main study hypothesis after finding support for our hypothesized factor model. Further, the variance extracted by the common method factor on which the items were loaded was 0.27, below the suggested criterion of 0.50 (Hair et al., 1998) and less than or comparable to the variance in other studies (e.g., Dulac et al., 2008, 0.29). These results demonstrated that common method variance in the present study was not serious.

**TABLE 1:** Results of confirmatory factor analysis.

Model’s	GFI	RMSEA	CFI	TLI	SRMR	X^2^	X^2^/df
Single factor model	0.54	0.37	0.39	0.45	0.41	637.12	8.45
Two factor model	0.63	0.31	0.47	0.63	0.36	443.86	6.23
Three factor model A	0.69	0.24	0.63	0.78	0.24	237.69	5.78
Three factor model B	0.79	0.15	0.75	0.86	0.18	211.32	3.28
Four factor model	0.91	0.05	0.91	0.90	0.04	152.42	2.56

Single-factor model: professional identity + critical thinking + leader motivational language + teacher success. Two-factor model: professional identity; critical thinking + leader motivational language + teacher success. Three-factor model A: professional identity; critical thinking + leader motivational language; teacher success. Three-factor model B: professional identity; critical thinking; leader motivational language + teacher success. Four-factor model: professional identity; critical thinking; leader motivational language; teacher success.

#### Descriptive statistics analysis

Table 2 displays the means, standard deviations, correlations, and scale reliability. All the study variables exhibited a satisfactory level of internal consistency, which was considered acceptable. The correlations between study variables were in the predicted direction, and all study variables had an acceptable degree of internal consistency (α > 0.70). The professional identity was positively related to critical thinking (*r* = 0.20, *p* < 0.01) and employee success (*r* = 0.31, *p* < 0.01).

**TABLE 2:** Mean, standard deviation, correlations and reliability.

Variables	Mean	SD	1	2	3	4	5	6	7	8
1. Gender	1.40	0.49								
2. Age	2.51	0.75	0.013							
3. Experience	1.81	0.81	0.060	0.013						
4. Education	2.74	0.46	–0.031	0.064	–0.046					
5. Professional identity (PI)	3.56	0.67	–0.002	0.051	0.051	0.329**	**(0.84)**			
6. Teacher success (TS)	3.74	0.54	–0.011	0.023	0.036	0.261**	0.315**	**(0.78)**		
7. Leader motivational language (LML)	3.22	0.77	0.007	0.026	–0.030	0.214*	0.475**	0.254**	**(0.83)**	
8. Critical thinking	3.66	0.78	0.010	0.041	0.023	0.319*	0.205**	0.421**	0.243**	**(0.81)**

*Correlation is significant at the 0.05 level (2-tailed). **Correlation is significant at the 0.01 level (2-tailed). Bold values indicate the Cronbach’s alpha.

#### Main effect and mediating effect analysis

Table 3 reports the hierarchical linear modeling (HLM) results for testing H1. After controlling for age, work experience professional identity had significant positive impacts on employee success (β = 0.037, *p* < 0.001, Model 4). Thus, professional identity can promote employees’ employee success, supporting H1. The mediation analysis has been done by using Hayes and Rockwood (2017) PROCESS macro model 4. Critical thinking mediates the link between professional identity and employee success (direct effect = 0.32, SE = 0.027, 95% CI = 0.2145, 0.3712; indirect effect = 0.43, SE = 0.044, 95% CI = 0.2586, 0.4394). Thus, it confirms H2.

**TABLE 3:** Hierarchical linear regression results.

	Critical thinking			Teacher success		
Variables	Model 1	Model 2	Model 3	Model 4	Model 5	Model 6
Age	0.04	0.03	0.02	0.03	–0.07	0.05
Gender	–0.02	0.04	0.01	0.01	–0.02	–0.04
Education	–0.03	0.01	–0.06	0.05	0.04	–0.01
Service	0.02	0.01	–0.07	–0.06	0.01	0.02
PL				0.037***		
**Mediator**						
Critical thinking					0.323***	
**Moderator**		–0.1712***				
LML						
**Interaction effect**						
PI × LML			–0.1348**			
*R* ^2^	31.56***	29.56***	25.61**	21.35**	26.62***	27.12***
*F*	0.33	0.15	0.11	0.17	0.20	0.26

**Correlation is significant at the 0.01 level (2-tailed). ***Correlation is significant at the 0.001 level (2-tailed).

#### Moderating effect analysis

**FIGURE 2:**
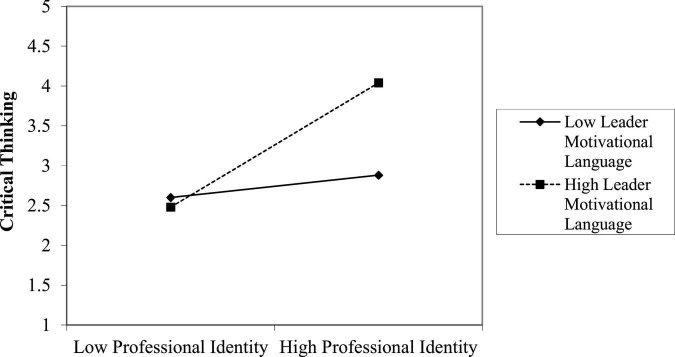
Moderation effect of leader motivational language.

For moderation analyses, we used Hayes (2017) PROCESS macro model 1. H3 stated that, leader motivational language significantly moderated the positive relationship between professional identity and critical thinking as shown in Table 3 (B = –0.1712^***^, SE = 0.06, *p* < 0.05, 95% CI = –0.2086, –0.0241). Figure 2 shows the interaction effect, which we displayed to make it easier to understand. Leader motivational language was measured using basic slopes tests (i.e., + 1 and –1 SD from the mean) (Aiken et al., 1991). Our findings demonstrated a favorable and substantial link between the professional identity and critical thinking when a leader motivational language was high (simple slope = 0.87, SE = 0.06, *p* < 0.001, 95% CI = 0.7745, 1.0653); as compare to when it is low (simple slope = 0.69, SE = 0.04, *p* < 0.001, 95% CI = 0.6441, 0.8466). The results confirm H3 hypothesis.

#### Moderation mediation examination

The moderation mediation analyses have been done by using Hayes (2017) PROCESS macro model 7. As suggested by Hayes (2017), we examined moderation mediation analysis of H3a, in an integrative way (One SD above and below the mean of a moderator) (Table 4). It was shown that the moderated mediated model, which included the outcome variable of employee success, was significant when leader motivational language was high (conditional indirect effect = 0.2824, SE = 0.0408, 95% CI = 0.2035, 0.3112), as compare to when leader motivational language is low (conditional indirect effect = 0.1916, SE = 0.0157, 95% CI = 0.1532, 0.245) as shown in Figure 2. As a result, the index of moderated mediation was shown to be significant (Index = –0.0366, SE = 0.0173, 95% CI = 0.067, 0.0147). Thus, confirms H3a hypothesis.

**TABLE 4:** Moderated mediated results for PI and TS across levels of LML.

Mediator	Level	Conditional indirect effect	SE	LLCI	ULCI
Critical thinking	Low	0.1916	0.0157	0.1532	0.2645
	High	0.2824	0.0408	0.2035	0.3112
	Difference	0.0908	0.0251	0.0503	0.0467

Moderator values are the mean and ± 1 SD, LLCI lower limit 95% confidence interval, ULCI upper limit 95% confidence interval.

## Discussion

The purpose of this study was to examine the association between employees’ professional identities, their critical thinking, and their level of success, and more particularly, to evaluate the roles that leadership motivating language plays in the success of employees. In response to the first study question, the findings of the correlational studies indicated a substantial and favorable relationship—one that runs in a positive direction—between the professional identity of workers and their level of success in their jobs (Zhang et al., 2018). In addition, critical thinking is a mediator between professional identity and employee success (Wang et al., 2020). This research also offers empirical evidence that the language of motivation used by leaders is a major factor in critical thinking and employee success. It seems from this that the language that leaders use may stimulate both the performance behavior and the thinking behavior of employees, which further enhances the success employees (Khan et al., 2021). Communication between leaders and followers may play an important role in developing successful employees. In addition, motivational language contributes to a better understanding of the psychological processes behind the antecedents of employee work behaviors (i.e., teacher success). This may pique more people’s interest in cultivating inspiring and communicative conduct among leaders and an effective conduit for workers’ critical thinking and success (Saeed et al., 2022).

### Theoretical implications

The outcomes of this study, which are supported by the literature, lead to a substantial theoretical contribution in the following way: The professional identity of employees makes a positive and significant contribution to employee success. In other words, the professional identities of employees play significant roles in the levels of success they achieve. The findings of this research are constructive and helpful to workers employed by Chinese MNC’s schools. According to the arguments expressed by Derakhshan et al. (2020) employees who cultivate their professional identities not only turn opportunities, but also promote achievement and success. In addition, employees should continually interpret and reinterpret their professional identities to become more effective in their job (Ruohotie-Lyhty, 2013; Khan et al., 2022b). Results of this study may also provide useful advice for training programs aimed at helping workers to create their professional identities. Programs like this should emphasize the value of professional identity to assist workers develop critical thinking skills. Second, professional identity enhances individuals’ ability to think critically. Consider both short-term objectives and long-term goals while practicing the skill of assessing one’s thought process. They assess what they have and identify what they will need to succeed. It is strongly suggested that employees develop their capacity for critical thinking to successfully handle hurdles to be a success, as well as to challenge internal and external barriers and increase ways that are socially acceptable to achieve success (Anika and Nurhayati, 2021). Thirdly, leaders know they should get better results from their team; what they rarely acknowledge is that they lack an important skill needed to make that happen. Managing is about motivating people to achieve at a higher level: accomplishing tasks, reaching goals, and achieving the organization’s vision (Wang et al., 2020). What inspires employees is well-crafted communication from their boss that is clear and transparent, that signals empathy for others, and that helps people derive meaning from their work (Kosmala and Herrbach, 2006). Language permeates a manager’s work life and can determine the success or failure of every communication a manager sends, be it an email, a project proposal, or a performance review (Jerez-Jerez and Melewar, 2020). In summary, Leaders pay attention to the expression and substance of their leadership speech, and they work to improve the meaning, affection, and direction of their leadership discourse. Three different aspects of a leader’s language are not interchangeable. They must be embraced in a coordinated way to achieve the greatest success inside an organization. To be more specific, the language that creates meaning and the language that provides direction urge individuals to contribute to the employee success (Pierrakos et al., 2009).

### Practical implication

Personal, social, and cultural aspects of professional identity building were shown to be intertwined in a thorough literature review. It was found that employee success was closely tied to the interplay between professional and vocation-related success as well as the interplay between personal, social, and cultural identity components. Create a professional identity for yourself so that you can take charge of your career. The results of this research provide organizations with ideas that may be put into practice to improve the effectiveness of employees. These suggestions focus on encouraging leaders to use motivating language and developing high-quality relationships with followers throughout the daily work process. First, the use of motivational language by leaders has been proven to be a successful management practice in corporate human resources work systems as well as a positive approach for generating followers’ professional identity. Given the significance of leader motivational language, it is imperative that businesses place a strong emphasis on the development of leadership language skills and work to enhance existing training programs. Second, training programs are required to take a holistic approach to all three aspects of language: directional language, meaning-making language, and empathic language. Training in these three languages can produce the greatest benefit for an organization’s overall efficiency and the success of its employees. This is due to the fact that each language type plays a distinct role in the communication that occurs between superiors and subordinates and that this role cannot be replicated using any other language type. Third, it is in an organization’s best interest to adopt a flat organizational structure that does away with all of the different levels of management in order to better serve its workforce. As a consequence of this, managers and workers are able to engage in two-way communication inside a flat organizational structure. This not only enhances the efficiency of communication but also assures that communication is clear and intelligible since there are no middlemen engaged in the process of communication to enhance their professional identity and success. Additionally, leaders should examine the possibility of installing a corporate social communication and collaboration platform.

### Future direction and limitation

Naturally, every study has its limitations, and the one being discussed here is no exception. One of the limitations of the current research is that it only had a limited number of participants in the sample. As a result, the current study may be repeated with a larger number of participants to ascertain whether or not the results will be the same or comparable to the previous ones. The next restriction is associated with the setting of the current investigation, which was conducted solely in China. As a result, it is important to proceed with caution when attempting to generalize the results. As a consequence, future study needs to be able to focus on the employee settings of multinational companies in various countries in order to investigate the possibility of variations in the outcomes. In addition, the current research made exclusive use of quantitative methods for both the collecting of data and the subsequent analysis of that data. To acquire more in-depth and all-encompassing conclusions from future research, it may be able to triangulate the data by integrating some interviews or focus groups in addition to the standard data collection methods.

## Data availability statement

The original contributions presented in this study are included in the article/supplementary material, further inquiries can be directed to the corresponding author/s.

## Author contributions

FD, XL, and AA contributed to the conception and design of the study. FD and XL organized the database and wrote the first draft of the manuscript. FA and ML performed the statistical analysis and wrote the sections of the manuscript. All authors contributed to the manuscript revision, read, and approved the submitted version.
