# Corrigendum: The link between English foreign language teacher's professional identity and their critical thinking that leads to teacher's success in the Chinese context: Leaders motivational language as a moderator

**DOI:** 10.3389/fpsyg.2022.1022176

**Published:** 2022-09-05

**Authors:** Fangfang Ding, Xingyu Liu, Alaa Amin Abdalla, Muhammad Latif Khan, Fouzia Akram

**Affiliations:** ^1^College of Humans, Sichuan Agricultural University, Ya'an, China; ^2^Academic Programs for Military Colleges, Abu Dhabi University, Al Ain, United Arab Emirates; ^3^Global College of Engineering and Technology, Muscat, Oman; ^4^Department of Business Administration, University of Prince Mugrin, Madina, Saudi Arabia

**Keywords:** professional identity, critical thinking, teacher success, leader motivational language, English Teacher, MNC's School

In the published article, there was an error in “affiliation 2.” Instead of “^2^Academic Programs for Military Colleges, Abu Dhabi University, Zayed City, United Arab Emirates,” it should be “^2^Academic Programs for Military Colleges, Abu Dhabi University, Al Ain, United Arab Emirates.”

The authors apologize for this error and state that this does not change the scientific conclusions of the article in any way. The original article has been updated.

## Publisher's note

All claims expressed in this article are solely those of the authors and do not necessarily represent those of their affiliated organizations, or those of the publisher, the editors and the reviewers. Any product that may be evaluated in this article, or claim that may be made by its manufacturer, is not guaranteed or endorsed by the publisher.

